# Supplementary figures and images for: Risk of prostate cancer in relatives of prostate cancer patients in Sweden: A nationwide cohort study
Source: PLoS Med. 2021 Jun 1;18(6):e1003616. doi: 10.1371/journal.pmed.1003616 (PMC8168897; doi:10.1371/journal.pmed.1003616)

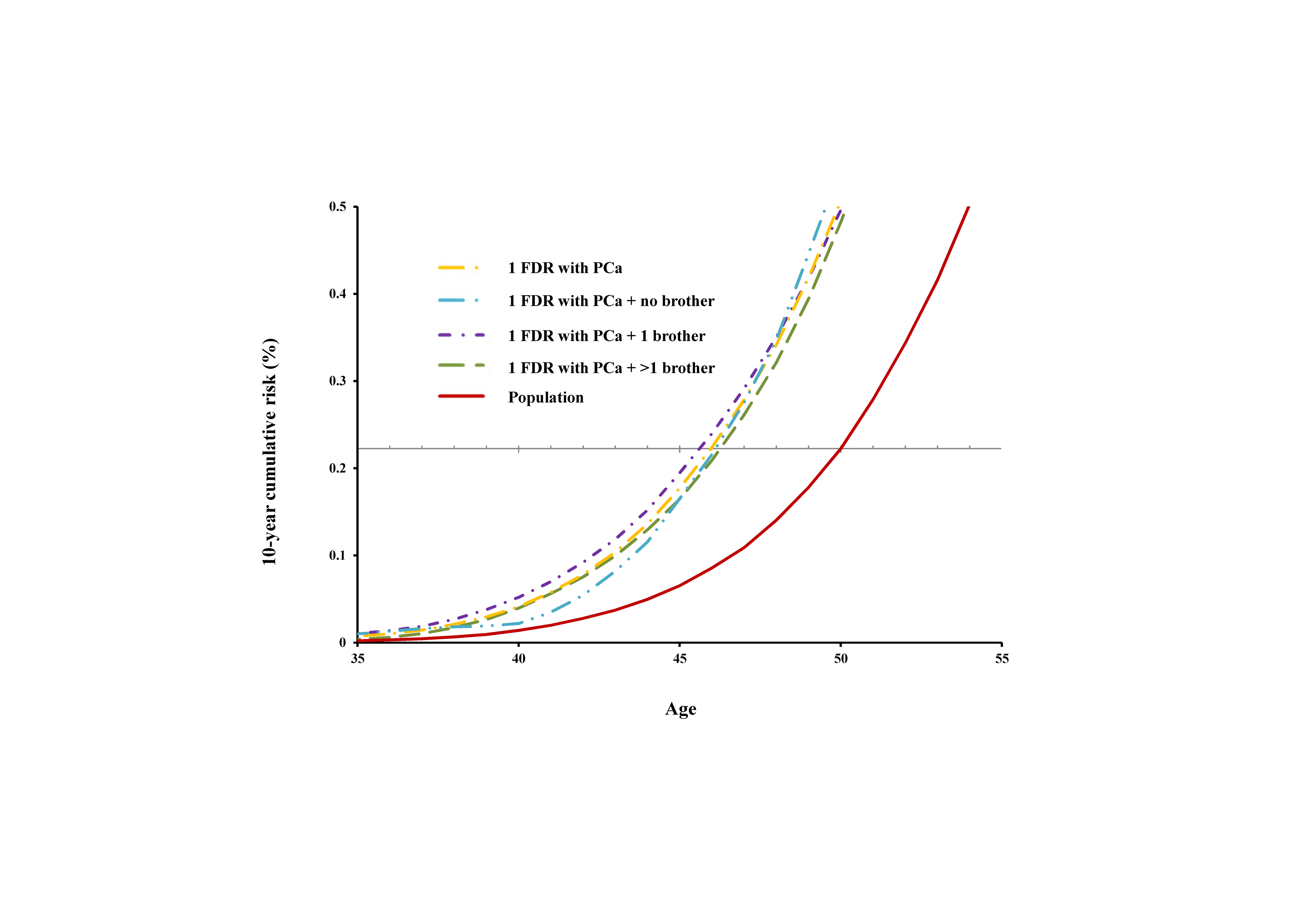

Supplement: S1 Fig — The gray horizontal line corresponds to 10-year cumulative risk level for 50-year-old men in the population. FDR, first-degree relative; PCa, prostate cancer. (TIF) [file pmed.1003616.s002.tif]

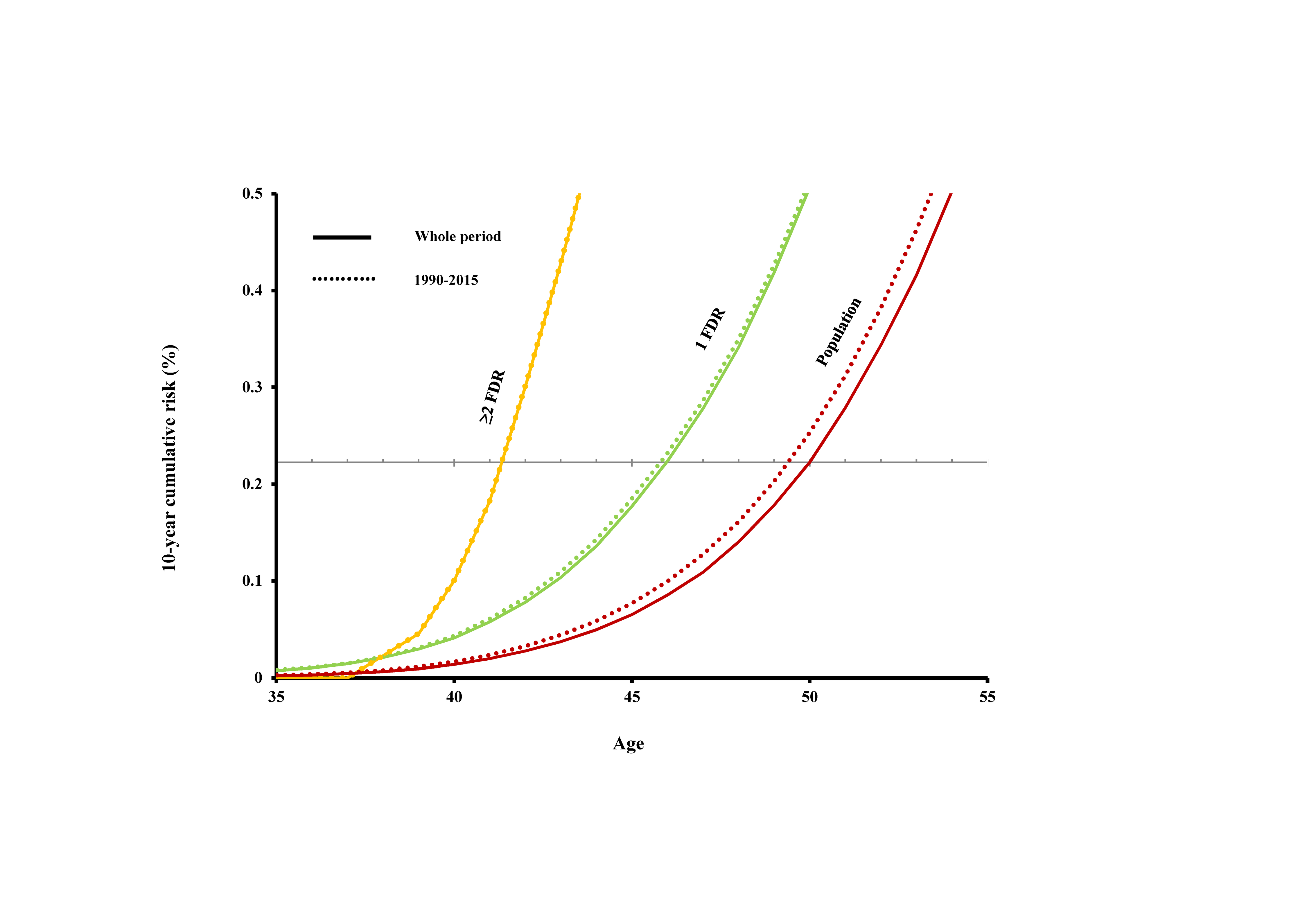

Supplement: S2 Fig — The gray horizontal line corresponds to 10-year cumulative risk level for 50-year-old men in the population. FDR, first-degree relative; PCa, prostate cancer. (TIF) [file pmed.1003616.s003.tif]
