# Supplementary material for: Risk of prostate cancer in relatives of prostate cancer patients in Sweden: A nationwide cohort study
Source: PLoS Med. 2021 Jun 1;18(6):e1003616. doi: 10.1371/journal.pmed.1003616 (PMC8168897; doi:10.1371/journal.pmed.1003616)
Supplement: S1 Table — PCa, prostate cancer. (DOCX) [file pmed.1003616.s004.docx]

**S1 Table. Risk-adapted starting age of prostate cancer screening for different benchmark starting ages of screening by type of affected relatives and age at diagnosis of the relative using 10-year cumulative risk**

| **Family history** | **Age at diagnosis of relative, y** | **Cancer patients (N)** |  | **Risk-adapted starting age of screening, y**  **(95% CI)** | | | |
| --- | --- | --- | --- | --- | --- | --- | --- |
| **Population [benchmark age]** | **-** | 88,999 |  | **[45]** | **[50]** | **[55]** | **[60]** |
| **Father** | **All ages** | 2,738 |  | 41 (41-42) | 46 (45-46) | 51 (51-51) | 56 (55-56) |
|  | **<60** | 182 |  | 39 (37-41) | 42 (41-44) | 47 (45-49) | 52 (50-53) |
|  | **60-69** | 675 |  | 41 (40-43) | 45*(44-46) | 50 (49-51) | 54 (53-55) |
|  | **≥70** | 1,881 |  | 42 (41-43) | 47 (46-47) | 52 (51-52) | 57 (56-57) |
| **1 brother** | **All ages** | 1,010 |  | 41 (40-43) | 44 (43-45) | 48 (46-49) | 52 (51-53) |
|  | **<60** | 263 |  | 41 (39-43) | 44 (42-45) | 47 (45-48) | 51 (49-53) |
|  | **60-69** | 591 |  | 42 (41-45) | 45*(43-48) | 49 (47-51) | 53 (51-54) |
|  | **≥70** | 156 |  | 48 (47-49) | 49 (48-50) | 53 (50-55) | 57 (53-60) |
| **10-year cumulative risk in the general population** | | |  | **0.1%** | **0.2%** | **0.6%** | **1.3%** |

Bold ages 45, 50, 55, and 60 indicate benchmark starting ages of prostate cancer screening in the general population.

*Example: When recommended benchmark starting age of prostate cancer screening in the general population was 50 years, men who had a history of prostate cancer diagnosed between age 60 and 69 only in one brother or only in his father attained the same risk level of 50-year-old men in the general population at age 45 and thus they could start screening five years earlier.
