# Supplementary material for: Risk of prostate cancer in relatives of prostate cancer patients in Sweden: A nationwide cohort study
Source: PLoS Med. 2021 Jun 1;18(6):e1003616. doi: 10.1371/journal.pmed.1003616 (PMC8168897; doi:10.1371/journal.pmed.1003616)
Supplement: S3 Table — FDR, first-degree relative; PCa, prostate cancer. (DOCX) [file pmed.1003616.s006.docx]

**S3 Table. Risk-adapted starting age of prostate cancer screening for different benchmark starting ages of screening by number of affected relatives and youngest age at diagnosis of first-degree relatives using 20-year cumulative risk**

| **Family history** | **Age at diagnosis of youngest relative, y** | **Cancer patients (N)** |  | **Risk-adapted starting age of screening, y**  **(95% CI)** | | | |
| --- | --- | --- | --- | --- | --- | --- | --- |
| **Population [benchmark age]** | **-** | 88,999 |  | **[40]** | **[45]** | **[50]** | **[55]** |
| **1 First-degree relative** | **All ages** | 3,576 |  | 36 (35-36) | 41 (41-41) | 46 (45-46) | 51 (50-51) |
|  | **<60** | 449 |  | 34 (31-35) | 38 (36-40) | 43 (42-45) | 49 (47-50) |
|  | **60-69** | 1,117 |  | 35 (34-36) | 40 (39-41) | 45*(44-45) | 50 (49-50) |
|  | **≥70** | 2,010 |  | 37 (36-37) | 42 (41-42) | 47 (46-47) | 52 (51-52) |
| **≥2 First-degree relatives** | **All ages** | 311 |  | 31 (30-35) | 35 (32-38) | 39 (37-41) | 43 (42-45) |
|  | **<60** | 120 |  | 31 (29-34) | 34 (31-37) | 39 (36-41) | 43 (41-45) |
|  | **≥60** | 191 |  | 33 (31-37) | 36 (34-39) | 40 (36-43) | 44 (41-47) |
| **20-year cumulative risk in the general population** | | |  | **0.2%** | **0.7%** | **1.5%** | **2.8%** |

Bold ages 40, 45, 50, and 55 indicate benchmark starting ages of prostate cancer screening in the general population.

*Example: When recommended benchmark starting age of prostate cancer screening in the general population was 50 years, men who had a history of prostate cancer diagnosed between age 60 and 69 only in one first-degree relative attained the same risk level of 50-year-old men in the general population at age 45 and thus they could start screening five years earlier.
