# Supplementary material for: Risk of prostate cancer in relatives of prostate cancer patients in Sweden: A nationwide cohort study
Source: PLoS Med. 2021 Jun 1;18(6):e1003616. doi: 10.1371/journal.pmed.1003616 (PMC8168897; doi:10.1371/journal.pmed.1003616)
Supplement: S1 Analysis Plan — (DOCX) [file pmed.1003616.s007.docx]

**S1 Analysis plan**

**1. Research question(s) and/or hypotheses**

Risk of aggressive prostate cancer in relatives of patients with this cancer in Sweden.

**2. Dataset(s) to be used**

Swedish family-cancer datasets including Swedish Cancer Registry, Multi-generation Register, national censuses, and Cause of Death Register in Sweden will be used. They were linked using a unique national identification number.

**3. Inclusion/exclusion criteria**

All men residing in Sweden who were born between 1932 and 2015 along with their parents will be included in this study. Parents’ ages are not limited but offspring are followed up to age 84 years.

**4. Variables to be used in the main analysis (the main exposure, outcome, and stratifying variables)**

Data on family relationships will be obtained from the Multi-generation Register. A 4-digit diagnostic code according to the 7th revision of the International Classification of Diseases (ICD-7) and subsequent ICD classifications are available in the Cancer Registry data from 1958 to 2015. The underlying cause of death is available from the Swedish Cause of Death Register.

The exposure of interest is having a history of PCa (regardless of TNM stage or cancer-specific death status) in first-degree relatives (FDRs: father, brother, or son). The outcome of interest is either diagnosis of stage III/IV PCa at the time of first diagnosis (based on the American Joint Committee on Cancer, 8^th^ edition of cancer TNM staging) and/or death due to PCa [1]. The death is considered due to PCa when PCa was reported as the main underlying cause of death. As data on prostate-specific antigen and Gleason score/grade group are not available in the Swedish Cancer Registry, in the analysis, only those with ‘T3-4, N0, and M0’ status will be considered as stage III prostate cancer. Those with ‘T1-2, N0, and M0’ status will not be considered as “with outcome” unless they died of PCa. Stage IV is defined as either ‘Any T, N1, and M0’ or ‘Any T, Any N, and M1’. The follow-up started at birth, immigration date, or starting date of the study, 1 January, 1958, whichever came latest. The follow-up ended on the year of PCa diagnosis, year of death, emigration or closing date of the study, 31 December 2015, whichever came earliest.

The dynamic definition of family history of PCa (and not the static approach) is used to consider the changes in family history over time. In brief, the dynamic family history of PCa in every participant is defined at entry into the study and it changes every time that a new family member is diagnosed with PCa. For instance, if there is an index man with two brothers who were diagnosed with prostate cancer in 1992 and 1998 and he himself was diagnosed with prostate cancer in 1995, under the dynamic definition of family history, the brother diagnosed in 1998 would not be counted into the family history of index man since in real-world scenario, one does not know the future history of their relatives. When a man has only one first-degree relative diagnosed with prostate cancer, he would be considered as men in group ‘1 FDR’. Once the second first-degree relative is diagnosed, he would shift to the group ‘>1 FDR’ until his own diagnosis of stage III/IV or fatal prostate cancer or the end of follow-up. This represents the actual change in the real-world when men mostly learn about the diagnosis of his close relatives at different time points of his life. For each year in the follow-up of individuals, the family history profile is updated for every index man, representing the real-time status (number and youngest age at diagnosis among relatives) of individuals with PCa diagnosed in his family. Occurrence of PCa in family members after the date of PCa diagnosis in the index patient will not be considered in familial risk calculations.

**5. Statistical methods and software to be used**

The 10-year cumulative risk is calculated based on the following equations [2]:

- *Age-specific annual incidence rate = Number of cases during each 1-year follow-up divided by person-years*
- *10-year cumulative rate for age X = Sum of 10 consecutive annual age-specific incidence rates from age X to age X+9*
- *10-year cumulative risk = 1 – exp ^(–10-year cumulative rate)^*

Exact values for person-years from individual data are used in the calculation of cumulative incidences. Risk-adapted starting ages of screening will be determined by the age at which men with different constellations of family history of PCa reached the same level of risk as men in the general population at benchmark ages 40, 45, 50, 55, or 60 years. The 95% confidence intervals of age-specific10-year cumulative risk will be calculated using the 2.5 and 97.5 percentiles of the bootstrap estimate distribution made by bootstrapping method (200 replications), from which confidence intervals of proposed starting ages (ages of reaching mass screening level of risk) will be derived according to the abovementioned formula for conversion of rate to risk.

The risk-adapted starting age of prostate cancer screening are based on the age at which the risk of stage III/IV and/or lethal PC reaches the same level as such risk for men at benchmark starting age of screening in the general population. A sensitivity analysis will be conducted to compare results for period 1990-2015 (after introducing PSA testing) and for the whole study period, 1958-2015. All analyses will be performed using SAS 9.4 (SAS Institute Inc., Cary, NC, USA). The study protocol was approved by the Lund Regional Ethics Committee (2012/795). Pseudonymized data will be used for analyses.

**6. Key table template**

**Example table. Risk-adapted starting age of prostate cancer screening for different benchmark starting ages of screening by number of affected first-degree relatives and youngest age at diagnosis of relatives using 10-year cumulative risk**

| **Family history** | **Age at diagnosis of youngest relative, y** | **Cancer patients (N)** |  | **Risk-adapted starting age of screening, y**  **(95% CI)** | | | |
| --- | --- | --- | --- | --- | --- | --- | --- |
| **Population [benchmark age]** | **-** |  |  | **[45]** | **[50]** | **[55]** | **[60]** |
| **1 First-degree relative** | **All ages** |  |  |  |  |  |  |
|  | **<60** |  |  |  |  |  |  |
|  | **60-69** |  |  |  |  |  |  |
|  | **≥70** |  |  |  |  |  |  |
| **≥2 First-degree relatives** | **All ages** |  |  |  |  |  |  |
|  | **<60** |  |  |  |  |  |  |
|  | **≥60** |  |  |  |  |  |  |
| **10-year cumulative risk in the general population** | | |  |  |  |  |  |

Bold ages 45, 50, 55, and 60 indicate benchmark starting ages of prostate cancer screening in the general population.

**References**

1. Amin MB, Edge S, Greene F, Byrd DR, Brookland RK, Washington MK, et al. AJCC Cancer Staging Manual. 8 ed: American Joint Committee on Cancer. Springer International Publishing; 2017. XVII, 1032 p.

2. Cancer registration: principles and methods. IARC Sci Publ. 1991(95):1-288.
