# Supplementary material for: Risk of prostate cancer in relatives of prostate cancer patients in Sweden: A nationwide cohort study
Source: PLoS Med. 2021 Jun 1;18(6):e1003616. doi: 10.1371/journal.pmed.1003616 (PMC8168897; doi:10.1371/journal.pmed.1003616)
Supplement: Attachment — Submitted filename: V2Review.docx [file pmed.1003616.s008.docx]

July, 2020

Review of manuscript PMEDICINE-D-20-02475R1

"Risk-adapted starting age of screening in relatives of patients with prostate cancer: Real world evidence from a nationwide cohort to supplement current screening recommendations"

**PLOS MEDICINE**

# GENERAL REMARKS

This study aimed to provide risk-adapted starting age of screening for relatives of patients with prostate cancer (PCa).

The authors analyzed a register-based nationwide cohort study, including all men residing in Sweden who were born after 1931 along with their parents. During the follow-up (1958-2015) the data of 6,343,727 men were assessed. The outcomes were defined as the diagnosis of stage III/IV PCa or death due to PCa, stratified by age at diagnosis.

It was observed:

1) 115,166 were diagnosed with stage III/IV PCa or died of PCa
2) Using 10-year cumulative risk curves, the authors calculated risk adapted starting ages of screening for men with different constellations of family history of PCa. (Family history being defined as a dynamic, time-dependent, variable considering changes during follow-up).
a. The 10-year cumulative risk of late-stage/fatal PCa in men at age 50 in the general population (a common recommended starting age of screening) was 0.2%.
b. Men with ≥ 2 FDRs (1^st^ degree relatives) diagnosed with PCa reached this screening level

- nine years earlier when the youngest one was diagnosed before age 50;
- seven years earlier when ≥ 2 FDRs were diagnosed after age 59, which was similar to men with one FDR diagnosed before age 60;
- and 3-5 years earlier when one FDR was diagnosed after age 59.

The authors concluded that:

- the study provides risk-tailored starting ages for PCa screening based on highly-valid nationwide data
- the findings, as an important step toward evidence-based personalized PCa screening guidance, can supplement current PCa screening guidelines for relatives of patients with PCa.

**EVALUATION**

The aim of the study is well defined. It is an interesting study dealing with the modalities of PCa screening for FDR in high risk families. It is known that family history is a significant risk factor for FDR increasing with the number of affected relatives in the family and early onset of the disease, this latter being a characteristic of inherited cancers. However, guidelines of screening in such families lack of precise recommendations according to the number of affected relatives and the onset of the PCa in the family. Moreover, as well underlined by the authors, screening in such families, and too early, may be associated with an increased risk of diagnosing indolent PCa and therefore overdiagnosis and overtreatment. It is the reason why the authors considered specifically the risk of late-stage/fatal risk of PCa which is a strength of the study. Using a large register-based nationwide cohort study, considered as the world’s largest nationwide register-based family-cancer datasets, authors were able to provide very relevant results i.e. the 10-year (and also 15-year and 20-year) cumulative risk of invasive PCa increased according the number of affected relatives and by the youngest age at diagnosis in the family. These results can be considered as evidence-based risk-adapted starting ages of screening for PCa.

The conclusions are in agreement with the results.

The title is appropriate and the abstract provides significant and concise information. References are relevant and cited in the manuscript.

This is a very relevant well written paper and only few modifications should be done:

The authors should propose at the end of the discussion clear practical guidelines, as they would like to be supported by the different urologic and oncologic societies, on how to screen FDR in high risk families according number of affected relatives and the youngest affected in the family

**In conclusion** this study can be published with minor corrections
