# Supplementary material for: Risk of prostate cancer in relatives of prostate cancer patients in Sweden: A nationwide cohort study
Source: PLoS Med. 2021 Jun 1;18(6):e1003616. doi: 10.1371/journal.pmed.1003616 (PMC8168897; doi:10.1371/journal.pmed.1003616)
Supplement: Attachment — Submitted filename: Response to reviewers 26Aug2020.docx [file pmed.1003616.s009.docx]

**Requests from the editors:**

*Within the Abstract Methods and Findings section, please include a brief note about any key limitation(s) of the study's methodology.

*Response: The following sentence was accordingly added to Abstract, Method and Findings section, last line*:

“No genetic data or information on lifestyle was available.”

*At this stage, we ask that you include a short, non-technical Author Summary of your research to make findings accessible to a wide audience that includes both scientists and non-scientists. The Author Summary should immediately follow the Abstract in your revised manuscript. This text is subject to editorial change and should be distinct from the scientific abstract. Please see our author guidelines for more information: <https://journals.plos.org/plosmedicine/s/revising-your-manuscript#loc-author-summary>

*Response: Following Author Summary was added immediately after the Abstract in our revised manuscript:*

**Author Summary**

**Why was this study done?**

- Family history is the strongest known risk factor for prostate cancer and current guidelines concur that an earlier screening for men with a family history of prostate cancer is necessary. However, limited evidence-based guidance is available on at what age actually this early screening should start.
- This study was conducted to provide precise recommendations about at what age should relatives of prostate cancer patients start screening based on the number of affected relatives and the age at onset of prostate cancer in the family.

**What did the researchers do and find?**

- In this nationwide study on 6,343,727 men, all men residing in Sweden who were born after 1931 along with their fathers were included.
- This study estimated the risk of late-stage or fatal prostate cancer in close family members of patients with prostate cancer.
- High-quality evidence on how many years earlier than the general population relatives of patients with prostate cancer could undergo screening was provided.
- When a man visits a clinic for advice on prostate cancer screening, the clinician can easily look up the table in this study and find the suitable starting age of screening in relatives of patients with prostate cancer based on two simple questions:

1) How many of your close relatives (father, brothers or sons) have ever received a prostate cancer diagnosis?

2) Was any of them diagnosed before age 60 years, or between 60 and 69 years?

**What do these findings mean?**

- This study using the world's largest data of its kind provided the best age for starting prostate cancer screening in the relatives of patients with prostate cancer.
- This study took into account not only the number of relatives but also age at onset of prostate cancer in the family members, which is additional important piece of information for the guidelines.
- The results may contribute to a more evidence-based personalized prostate cancer screening guidance in real-world settings and clinicians could inform patients with prostate cancer about this possibility and encourage individualized counselling for their relatives.

*Please clarify if the analytical approach followed in this study corresponded to one laid out in a prospective protocol or analysis plan. Please state this (either way) early in the Methods section.

a) If a prospective analysis plan (from your funding proposal, IRB or other ethics committee submission, study protocol, or other planning document written before analyzing the data) was used in designing the study, please include the relevant prospectively written document with your revised manuscript as a Supporting Information file to be published alongside your study, and cite it in the Methods section. A legend for this file should be included at the end of your manuscript.

b) If no such document exists, please make sure that the Methods section transparently describes when analyses were planned, and when/why any data-driven changes to analyses took place.

c) In either case, changes in the analysis-- including those made in response to peer review comments-- should be identified as such in the Methods section of the paper, with rationale.

*Response:* “The analyses were planned ahead and no data-driven changes to analyses took place. Some new analyses were conducted as requested by reviewers (e.g., 95% confidence intervals, comparison of results for period 1990-2015 with the ones for the whole study period, and risk of prostate cancer for men with one affected FDR who had 0/1/more brothers without prostate cancer).”

*We'd recommend the authors use an appropriate reporting guideline to support reporting of their study - one such guideline may be the RECORD guideline (https://www.equator-network.org/reporting-guidelines/record/) - designed for routinely collected health data. If using this guideline please cite the RECORD paper in the Methods section and also upload a completed RECORD checklist as supporting information with your revision.

*Response: A completed RECORD checklist was uploaded as supporting information with our revision. The use of RECORD guideline was mentioned in the Methods section, paragraph 5, line 7:*

“This study is reported as per The REporting of studies Conducted using Observational Routinely-collected health Data (RECORD) guidelines (1).”

**Comments from the reviewers:**

Reviewer #1 (Peter Flom):

I confine my remarks to statistical aspects of this paper. These were well done and I have only a couple of minor corrections/suggestions.

* p 3 Give the death rate for prostate cancer. "5th leading cause ...." is pretty meaningless.

*Response: Thank you for your comments. The sentence (Introduction, first paragraph, line 1) was revised to:*

“Prostate cancer (PCa) is the second most common cancer in men (age-standardized incidence rate 29.3 per 100,000) and a major cause of cancer deaths (age-standardized mortality rate 7.6 per 100,000) in the world.”

* p. 5 First para. Men don't necessarily learn about a relative's PC diagnosis.

*Response: In this study, the focus was put on close relatives (father, brother, and son) whose critical disease information could be most probably (but of course not necessarily) shared easily and timely. The sentence in Methods section, paragraph 3, line 13, was modified accordingly to*:

“This represents the actual change in the real-world when men mostly learn about the diagnosis of his close relatives at different time points of his life.”

* p. 5 Please format the formulas better, maybe separating them from the text.

*Response: The format of following sentences was accordingly modified in Methods, paragraph 4, line 2*:

- “The 10-year cumulative risk was calculated based on the following equations:
- *Age-specific annual incidence rate = Number of cases during each 1-year follow-up divided by person-years*
- *10-year cumulative rate for age X = Sum of 10 consecutive annual age-specific incidence rates from age X to age X+9*
- *10-year cumulative risk = 1 – exp ^(–10-year cumulative rate)^*”

Reviewer #2: Manuscript#: PMEDICINE-D-20-02475R1

Title: Risk-adapted starting age of screening in relatives of patients with prostate cancer: Real world evidence from a nationwide cohort to supplement current screening recommendations.
Criteria PLOS Medicine (website consulted on 04.07.2020):
General:
1. The research question is an important one to the community of researchers in this general area.
2. The results provide a substantial advance over existing knowledge, with clear implications for patient care, public policy, or clinical research agendas.
3. Published together with an Author Summary written for general readers, the article is of interest to clinicians and policymakers who are not specialists in this topic.
* What are the main claims of the paper and how significant are they for the discipline?
* Are the claims properly placed in the context of the previous literature? Have the authors treated the literature fairly?
* Do the data and analyses fully support the claims? If not, what other evidence is required?
* PLOS Medicine encourages authors to publish detailed methods as supporting information online. Do any particular methods used in the manuscript warrant such publication? If a protocol is already provided, for example for a randomized controlled trial, are there any important deviations from it? If so, have the authors explained adequately why the deviations occurred?
* Is this paper outstanding in its discipline? If yes, what makes it outstanding? If not, why not?
* Does the study conform to any relevant guidelines such as CONSORT, MIAME, QUORUM, STROBE, and the Fort Lauderdale agreement?
* Are details of the methodology sufficient to allow the experiments to be reproduced?
* Is any software created by the authors freely available?
* Is the manuscript well organized and written clearly enough to be accessible to non-specialists?

Summary:

In the revised manuscript the authors analyze the complete Swedish men population in a longitudinal observation between 1958 and 2015 with the goal to study the relatives having had advanced PCa or who succumbed to PCa for purpose of calculating the risk-adapted starting age of screening for PCa for their relatives. They calculate the 10-year cumulated PCa risk in the whole population (0.2%) as a reference cutoff value and test it against various populations build on criteria of number of relatives with PCa and age of PCa diagnosis. They demonstrate that these populations reach this risk cutoff at the different age and thus they propose tailoring the onset of PCa screening accordingly.
This topic is very relevant for everyday practice of every clinician dealing with early detection of PCa, thus all three general criteria of PLOS Medicine as listed above are fulfilled.
As the authors properly describe in the introduction - a substantial body of literature exists on the topic and there is a clear agreement that positive family history increases risk of having PCa in significant manner, thus these men should receive more screening for PCa and at earlier age. However, the proposed cutoffs vary and if you consult medical guidelines (e.g. EAU or ESMO) these cutoffs can be perceived as an extrapolated and rather intuitive best guess concerning the starting age of screening. With this important study the authors try to fill this gap in more precise manner. Moreover they take into account not only the number of relatives but also onset of disease in those relatives, which is additional important piece of information.

Comments

* Introduction

Is well written. No relevant remarks.

*Response: Thank you.*

* Methods

Material for the study is well chosen and we can in general commend our Swedish colleagues for the quality registry data which have been collected. The statistical methods seem adequate and sufficient, at least at the understanding of reviewer who is clinician but with experience dealing with epidemiological and statistical methods and data. Still as the reviewer is not specialist in statistics a statistical review should be warranted. I would suggest to make a reference(s) to method source (can be a paper or manual) for the reader for better understanding.

*Response: Thank you for your suggestion. The following reference (2) was added to Methods section, paragraph 2, line 1:*

“Jensen OM et al. Cancer registration: Principles and methods. IARC Sci Publ. 1991(95):1-288.”

* While the choice of the study event (advanced PCa or PCa death) seems correct as for purpose of the study some unclarities remain. Men who die at PCa are usually having PCa diagnosis before this event. Do I understand correctly that for men diagnosed with advanced PCa but dying from other causes this event was taken for study? It should be clarified as PCa diagnosis may be at advanced or local stage and then progress to PCa death (or not). Wouldn't it be more appropriate and consistent to take stage shift (or diagnosis) at advanced PCa stage as study event?

*Response: PCa-specific death was defined as one of the main outcomes, which means we considered dead cases as ‘with outcome’ when PCa was the main cause of death. Furthermore, men with PCa who died due to other causes were excluded unless they had an advanced stage (III/IV) at the time of initial PCa diagnosis. For both those who were diagnosed with advanced PCa and those later died from PCa, the time of event started from the time of PCa diagnosis. Only stage status at the time of first diagnoses were recorded in our database and thus we had no data on stage shift. The following sentence was accordingly modified in Methods, paragraph 2, line 7*:

“The outcome was either diagnosis of stage III/IV PCa at the time of first diagnosis (based on the American Joint Committee on Cancer, 8^th^ edition of cancer TNM staging) and/or death due to PCa (3).”

* For purpose of estimating the analysis reliability a reference (if available) to the reliability and completeness of both ascertainment of PCa death and disease stage in the registry should be done.

*Response: Two new references were added and following sentence was accordingly added to the Methods section, paragraph 1, line 11*:

“The Swedish Cancer Registry and Cause of Death Register have been reported to have high level of completeness and accuracy (4, 5).”

* It is important that authors also calculated the 15- and 20-year cumulative risks for having PCa as this adds to the robustness of the results.

*Response: Thank you. We added this point to strengths of our study in Discussion, paragraph 8, last sentence as follows:*

“Moreover, we also calculated the 15- and 20-year cumulative risks of advanced PCa with consistent estimates for starting ages, which adds to the robustness of our results.”

* Discussion

Is generally well written but feels somehow uncritical. The authors see all but study strengths and omit the limitations. I have several remarks.

"Nevertheless, it would be optimal when risk-adapted starting ages of screening in other countries with different races than Sweden for men with a family history of prostate cancer are externally validated although the current experts' opinion-based recommendations for screening relatives of patients with prostate cancer are already in practice without validation."

This sentence is difficult to follow. As I understand the authors perceive their data as kind of validation or reference dataset. But from that perspective even if this may go with our intuition still their data have not been externally validated in another population. It is particularly important as PCa mortality in Sweden is particularly high as compared to the whole western world. Thus what happens in Sweden does not necessarily have to be happening elsewhere. The reference to "different races than Sweden" is also unclear. The reviewer presumes (but may be wrong) that the caucasian men is the predominant race in Sweden and that results apply to this race only. This paragraph should be elaborated.

*Response: Following sentences were accordingly modified to Discussion section, paragraph 5, line 13:*

“Nevertheless, it would be optimal for the risk-adapted starting ages of screening to be externally validated in countries with population compositions different from Sweden although the current expert opinion-based recommendations for screening in relatives of patients with PCa are already in practice in many countries around the world without validation.”

* Study limitations are not mentioned. As already stated in previous comment, Swedish population has relatively high PCa specific mortality. While the methods used by the authors and the dynamic definition of positive family history may be generalized for other populations, the results of this study as such and the risk cutoffs may vary and might not be transferable to other countries or races.

*Response: To increase the generalizability of the results, we provided risk-adapted starting ages of screening for populations with different benchmark ages of initial mass screening (i.e. different risk levels), which can be used in countries with other benchmark ages than 50 years. Following sentences were accordingly added to Discussion, paragraph 9, last sentence:*

“Risk-adapted starting ages of screening for populations at different benchmark starting ages of screening were provided to fit in with countries that have different risk levels of PCa.”

*Discussion section, paragraph 5, line 13:*

“Nevertheless, it would be optimal for the risk-adapted starting ages of screening to be externally validated in countries with population compositions different from Sweden although the current expert opinion-based recommendations for screening in relatives of patients with PCa are already in practice in many countries around the world without validation.”

* Another obvious limitation derives inherently from the fact that in the study only 4.3% of all men with PCa fulfilled their dynamic definition of family history and formed the study population. Thus the most men doesn't have any family history.

*Response: Following sentences were accordingly modified/added to Discussion section, paragraph 2, last sentence:*

“However, considering a large proportion of men for whom the family history-based risk-adapted screening does not apply, further investigation by incorporating additional information on common variants and other risk factors is important to improve PCa screening and prevention in a more personalized manner.”

* PCa diagnosis changed in time, while DRE and autopsy were method of choice at the study beginning, other modalities like PSA (and it's derivates or other biomarkers) , ultrasound, mpMRI, etc. This might have influenced the events distribution.

*Response: Additional analysis by restricting the follow-up to period at/after year 1990 (the era of using PSA test for prostate cancer screening, results of which are more applicable to current time) was conducted and the risk estimations were consistent. Following sentences were accordingly modified/added to Methods, Results, and Discussion sections:*

*Methods, paragraph 5, line 3*:

“A sensitivity analysis was conducted to compare results for period 1990-2015 (after introducing PSA testing) and for the whole study period, 1958-2015.”

*Results, Sensitivity analysis subsection, paragraph 1, line 1:*

***“Sensitivity analysis***

A sensitivity analysis by calendar period at diagnosis comparing the results for the period 1990-2015 to the ones for the whole period (1958-2015) was conducted and the analysis showed consistent results (S2 Fig).”

*Discussion, paragraph 4, line 6:*

“It is possible that the age at diagnosis of cancer in family members could be affected by PSA screening, for which we had no data. No national population-based PSA screening program has ever been recommended in Sweden and organized PSA testing was implemented in some parts of Sweden only since 2019 (6). Despite this, an additional sensitivity analysis was performed to compare risk estimations over different periods and the results were consistent. A recent study has shown that even family history of prostate borderline or in situ carcinoma (mostly screen-detected lesions) and their age at diagnosis are rather equally important and should be taken into account in counselling patients and their relatives (7).”

* It is also important to convey to the reader that you can transfer these results only if you perform elaborate anamnesis of family history. A simple - "how many relatives if any had PCa in family" would be insufficient for that purpose. Has it been investigated how practicable and reliable is this approach in everyday GP practice? Checking register-based family history vs patients' self-reports would be interesting.

*Response: We agree with you. The results of this study are based on a nationwide highly valid register-based information, so the family histories that have been used for our estimates were precise and accurate. Of course, such kind of approach is more reliable than self-report family history, which is prone to information and recall bias. In order to benefit from results of this study family history should be asked in more details. Following sentences were accordingly added to Author Summary, Methods, Results, and Discussion sections:*

*Author Summary, paragraph2, bullet point 4:*

“When a man visits a clinic for advice on prostate cancer screening, the clinician can easily look up the table in this study and find the suitable starting age of screening in relatives of patients with prostate cancer based on two simple questions:

1) How many of your close relatives (father, brothers or sons) have ever received a prostate cancer diagnosis?

2) Was any of them diagnosed before age 60 years, or between 60 and 69 years?”

*Discussion, paragraph10, line 1:*

“A rather elaborate anamnesis of family history is important in implementing the findings of this study into clinical practice. A simple question "How many relatives if any had PCa in family?" would be insufficient for this purpose. As shown by our data, the higher number and the younger age at diagnosis of affected FDRs are both important factors associated with increased risk of aggressive/fatal PCa. A pilot test of oncological practices showed that complete family history report rates of 77% in FDRs and 61% in second-degree relatives, but age at diagnosis was recorded in only one-third of them (8). In order to benefit from results of this study, detailed family history of close relatives (father, brother, and son) would be needed along with the age at diagnosis of PCa in them (<60, 60-69, ≥70 years), which is of course easier to obtain than the exact age at diagnosis for each affected family member.

The American Society of Clinical Oncology guideline also recommends that emphasis on careful documentation of the family history of close relatives (9). Self-reports can still serve as an important source of information in clinical counselling since only information of close relatives would be needed. For countries with mature registry (or electronic health record) system like Sweden, family history could be technically taken from register-based datasets although to our knowledge this is not the case in any country. A recent practice improvement project at Mayo Clinic showed that enhanced family history screening was a low-cost but effective method for individualized cancer surveillance (10). Various tools for family history collection are being developed and systematic reviews have shown a 46-78% improvement in data collection (11, 12). As an attempt to alleviate the burden of physicians, health policy makers could remove the system barriers and facilitate the development and implementation of family history collection tools (e.g., by providing websites, software and/or apps to collect the necessary family history before the actual visit of men to the physician). Adequate health education to the lay population seems also very important for timely assessment of risk profile. Health economic studies are warranted to encourage insurances to cover cost of evidence-based personalized early screening in high-risk men.”

*Discussion, second last paragraph, line 3:*

“Country-specific clinical guidelines need to recommend men to start prostate cancer screening at different ages based on their family history and age at diagnosis of prostate cancer in their families. Results of our study (summarized in Table 1) can be easily used by clinicians based on answers to following two simple questions:

1) How many of your close relatives (father, brothers or sons) have ever received a prostate cancer diagnosis?

2a) If the answer to first question is 1, then ask:

Was the relative diagnosed before age 60 years, between 60 and 69, or at 70 or older?

2b) If the answer to first question is 2 or more, then ask:

Was any of relatives diagnosed before age 60 years?

”

* Minor remarks

"In general, our evidence-based results"

these results may become evidence if published thus "In general, our results" or "Our results" - whatever preferable.

*Response: This sentence was revised to:*

"In general, our results based on high-quality data …."

* "Although the incidence rate of prostate cancer varies by geography and race/ethnicity, the familial risks of cancers are generalizable across populations with approximately similar cancer incidence and pattern."

Any reference to this general statement?

*Response: The missing reference has been added to that sentence in Discussion, paragraph 5, line 9:*

“Although the incidence rate of PCa varies by geography and race/ethnicity, the familial risks of cancers are generalizable across populations with approximately similar cancer incidence and pattern (13).”

* Conclusion

The conclusion generalizes too far on "relatives of patients with PCa" as the authors explicitly chose advanced or lethal PCa as their study event. This self-limits the conclusions.

*Response: The exposure of interest was having first-degree relative(s) with PCa (regardless of stage or cancer-specific death status). The outcome was either diagnosis of stage III/IV PCa or death due to PCa. It could not be 100% correct if we would have claimed risk-adapted starting age of screening for relatives of patients with advanced PCa or those with family members died of PCa. To avoid such a misunderstanding by readers, we modified the following sentence in the Methods section, paragraph 2, line 5:*

“The exposure of interest was having a history of PCa (regardless of TNM stage or cancer-specific death status) in first-degree relatives (FDRs: father, brother, or son). The outcome was either diagnosis of stage III/IV PCa at the time of first diagnosis (based on the American Joint Committee on Cancer, 8^th^ edition of cancer TNM staging) and/or death due to PCa (3). The death was considered due to PCa when PCa was reported as the main underlying cause of death.”

*We also limited “relatives of patients with PCa” to “first-degree relatives of patients with prostate” in the conclusion sentence as follows:*

“In conclusion, this study provides novel information that can be used for risk-adapted starting age of screening in first-degree relatives of patients with PCa based on the world’s largest nationwide register-based family-cancer datasets.”

Reviewer #3: Comments

* The aim of the study is well defined. It is an interesting study dealing with the modalities of PCa screening for FDR in high risk families. It is known that family history is a significant risk factor for FDR increasing with the number of affected relatives in the family and early onset of the disease, this latter being a characteristic of inherited cancers. However, guidelines of screening in such families lack of precise recommendations according to the number of affected relatives and the onset of the PCa in the family. Moreover, as well underlined by the authors, screening in such families, and too early, may be associated with an increased risk of diagnosing indolent PCa and therefore overdiagnosis and overtreatment. It is the reason why the authors considered specifically the risk of late-stage/fatal risk of PCa which is a strength of the study. Using a large register-based nationwide cohort study, considered as the world's largest nationwide register-based family-cancer datasets, authors were able to provide very relevant results i.e. the 10-year (and also 15-year and 20-year) cumulative risk of invasive PCa increased according the number of affected relatives and by the youngest age at diagnosis in the family. These results can be considered as evidence-based risk-adapted starting ages of screening for PCa.

The conclusions are in agreement with the results.

The title is appropriate and the abstract provides significant and concise information. References are relevant and cited in the manuscript.

This is a very relevant well written paper and only few modifications should be done:

The authors should propose at the end of the discussion clear practical guidelines, as they would like to be supported by the different urologic and oncologic societies, on how to screen FDR in high risk families according number of affected relatives and the youngest affected in the family.

*Response: Thank you for your comments. Following sentences on clear practical guidelines were accordingly added to Discussion section, 2nd last paragraph, line 1*:

“The risk-adapted starting age of PCa screening for men with a family history of PCa ranged from three to 12 years earlier than men in the general population according to the number of affected FDRs and youngest age at diagnosis of relatives. Clinician can inform patients with PCa about the possible earlier screening in their first-degree relatives. Patients can encourage their close relatives to seek early counselling with their doctor to find out the appropriate age of initial PCa screening for them based on their specific detailed family history. Country-specific clinical guidelines need to recommend men to start PCa screening at different ages based on their family history and age at diagnosis of PCa in their families. Results of our study (summarized in Table 1) can be easily used by clinicians based on answers to following two simple questions:

1) How many of your close relatives (father, brothers or sons) have ever received a prostate cancer diagnosis?

2a) If the answer to first question is 1, then ask:

Was the relative diagnosed before age 60 years, between 60 and 69, or at 70 or older?

2b) If the answer to first question is 2 or more, then ask:

Was any of relatives diagnosed before age 60 years?

Of course, if the family history is going to be used for future research, it would be ideal to record exact number of affected relatives, their ages at diagnoses, and calendar years of their diagnoses when possible (14). As an example, when recommended benchmark starting age of prostate cancer screening in the general population in a country is 50 years, men who had one FDR diagnosed with PCa before age 60 could start screening at age 43, i.e. seven years earlier than his counterparts in the general population. If there were two FDRs with PCa in the family of that man, one diagnosed before age 60 and the other diagnosed after age 60, the youngest age at diagnosis would be before age 60 and therefore the man could be screened at age 39, i.e. 11 years earlier.”

Reviewer #4: Comments

* This paper uses data from Swedish national registries to compute risk of aggressive prostate cancer (PCa) based on family history of PCa. Examining general population risk and risk for various family history profiles, the paper determines ages of starting screening that have equivalent risk of aggressive prostate cancer for those with a given family history as compared to the general population. More context is needed about the level of PSA screening in Sweden during the analysis period, since this could effect the various estimates. Also, more discussion about the practicalities of family history risk-based screening are needed.

*Response: Thank you for your comments. Additional analysis by restricting the follow-up to period after year 1990 (the era of using PSA test for prostate cancer screening, results of which are more applicable to current time) was conducted and the risk estimations were consistent. Following sentences were accordingly added to Methods, Results, and Discussion sections:*

*Methods, paragraph 5, line 3*:

“A sensitivity analysis was conducted to compare results for period 1990-2015 (after introducing PSA testing) and for the whole study period, 1958-2015.”

*Results, Sensitivity analysis subsection, paragraph 1, line 1:*

**“*Sensitivity analysis***

A sensitivity analysis by calendar period at diagnosis comparing the results for the period 1990-2015 to the ones for the whole period (1958-2015) was conducted and the analysis showed consistent results (S2 Fig).”

*Discussion, paragraph 9, line 6:*

“Sweden with PCa mortality higher than many other developed countries is not considered as a highly-screened country (15). PSA testing prevalence has previously been shown to vary across different regions of Sweden (16, 17). Data from Stockholm region showed that, during 2010 and 2011, 25%, 40%, and 46% of men aged 50–59, 60–69, and 70-79 had a PSA test, respectively, whereas less than 20% of the male population underwent PSA testing in a large health care region in Sweden (17, 18). ”

*Discussion, paragraph 4, line 8:*

“No national population-based PSA screening program has ever been recommended in Sweden and organized PSA testing was implemented in parts of Sweden only since 2019 (6). Despite this, an additional sensitivity analysis was performed to compare risk estimations over different periods and the results were consistent. A recent study has shown that even family history of prostate borderline or in situ carcinoma (mostly screen-detected lesions) and their age at diagnosis are rather equally important and should be taken into account in counselling patients and their relatives (7).”

Specific Comments

* 1. In Methods, the benchmark ages are given as 40,45,50 and 55. However, in Table 1, the benchmark ages are 45,50, 55 and 60.

*Response: When 10-year cumulative risks were considered, the benchmark ages were given as 45, 50, 55, and 60. Only for cumulative risk of longer follow-up (15-year and 20-year), the benchmark ages in the tables in Supporting Information were given as 40, 45, 50, and 55, 5 years earlier to be comparable with those for 10-year cumulative risk. Although the core benchmark ages 45, 50, and 55 have been provided for all, following sentences were accordingly modified in Methods, paragraph 4, line 8*:

“Risk-adapted starting ages of screening were determined by the age at which men with different constellations of family history of PCa reached the same level of risk as men in the general population at benchmark ages 40, 45, 50, 55, or 60 years.”

* 2. Are there data on number of brothers? If so, is there any difference in risk for, say, a man with 1 first degree relative with PCa, if they had no brothers versus 1+ brothers (or 2+, etc.). More generally, could the exposure be refined based on number of male first degree relatives, in addition to how many had PCa.

*Response: It has been reported before that the number of relatives (family size) does not affect PCa risk (19). Additional analysis was done to compare the risk of PCa for men with brother unaffected with PCa and the results showed that the number of unaffected brothers did not change the risk estimations. S2 Fig was added to supplementary information and following sentences were added to Results and Discussion sections:*

*Results, paragraph 2, line 8:*

“An additional analysis was done to compare the risk of PCa for men with one affected FDR who had 0/1/more brothers without PCa and the results showed that the number of unaffected brothers does not change our results substantially (S1 Fig).”

*Discussion, paragraph 3, line 10:*

“It has been reported before that the number of relatives (family size) does not affect PCa risk (19). However, an additional analysis was conducted to compare the risk of PCa for men with one affected FDR who had 0/1/more brothers without PCa diagnosis and the results showed similar estimated risk-adapted starting ages of screening by number of unaffected brothers.”

* 3. A potential harm of earlier start of screening is being diagnosed earlier with an indolent cancer and suffering from side-effects of treatment longer or having to undergo active surveillance longer. This should be mentioned in the discussion, in addition to harms from extra false positives that are mentioned in Discussion.

*Response: The following sentence was accordingly added to Discussion, paragraph 2, line 19:*

“Any screening recommendations must consider the false positives and potential harms from screening individuals earlier. Side effects of longer treatment and extended active surveillance associated with earlier screening should be also taken into account.”

* 4. In order to interpret these data, it would be helpful to know the level of PSA screening in Sweden during the period of assessment. Presumably, it was low overall during the period linked to the cancer registry (1958-2015). The level of screening would certainly affect the proportion of men with a family history, which in turn would affect the proportion of men having to be screened at earlier ages. It could also affect the excess risk of stage III/IV or fatal cancer associated with a family history, since with screening a higher proportion of cases in relatives would be indolent, and indolent presentation could be in part genetically modulated. Thus the excess risk of stage II/IV or fatal disease associated with a family history could be lower in a screened population. This should be discussed.

*Response: The Swedish National Board of Health and Welfare does not recommend a countrywide population-based PSA screening program for men (20).* *Following sentences were accordingly modified in/added to Methods, Results, and Discussion sections:*

*Methods, paragraph 5, line 3*:

“A sensitivity analysis was conducted to compare results for period 1990-2015 (after introducing PSA testing) and for the whole study period, 1958-2015.”

*Results, paragraph 1, line 1:*

***“Sensitivity analysis***

A sensitivity analysis by calendar period at diagnosis comparing the results for the period 1990-2015 to the ones for the whole period (1958-2015) was conducted and the analysis showed consistent results (S2 Fig).”

*Discussion, paragraph 9, line 8:*

“PSA testing prevalence has previously been shown to vary across different regions of Sweden (16, 17). Data from Stockholm region showed that, during 2010 and 2011, 25%, 40%, and 46% of men aged 50–59, 60–69, and 70-79 had a PSA test, respectively, whereas less than 20% of the male population underwent PSA testing in a large health care region in Sweden (17, 18). The stage III/IV or lethal familial risk estimations could have been diluted when men with family history of PCa were diagnosed early due to actively seeking earlier PSA screening and did not die of PCa compared to men without family history, although a previous study has reported that familial aggregation of PCa is not due to screening habits shared in a family (21). Screening practice may change the reference group in calculation of familial relative risk of aggressive PCa (e.g., in terms of standardized incidence ratio). However, our results in terms of cumulative risk of aggressive PCa that has no reference group is not affected by screening practice and this explains the stable results over different periods in a sensitivity analysis that we conducted. The possible risk-adapted starting ages of screening for populations at different benchmark starting ages of screening were provided to fit in with countries that have different risk levels of PCa.”

*5. The practicalities of implementing screening based on family history need to be discussed more. What is the expense and patient and physician time spent on evaluating family history, especially if it needs to be updated periodically? In most countries, family history data would not be available from national registries. How could EHR systems handle these data and could simple algorithms be developed to determine family history-based risk levels? Is there the potential for confusion in the population of the appropriate starting age if guidelines are based on family history, rather than a simple standard age?

*Response: In current guidelines, there have already been recommendations for PCa screening based on family history, though without detailed information. Following sentences were accordingly added:*

“

*Author Summary, paragraph2, bullet point 4:*

“When a man visits a clinic for advice on prostate cancer screening, the clinician can easily look up the table in this study and find the suitable starting age of screening in relatives of patients with prostate cancer based on two simple questions:

1) How many of your close relatives (father, brothers or sons) have ever received a prostate cancer diagnosis?

2) Was any of them diagnosed before age 60 years, or between 60 and 69 years?”

*Discussion, paragraph10, line 1:*

“A rather elaborate anamnesis of family history is important in implementing the findings of this study into clinical practice. A simple question "How many relatives if any had PCa in family?" would be insufficient for this purpose. As shown by our data, the higher number and the younger age at diagnosis of affected FDRs are both important factors associated with increased risk of aggressive/fatal PCa. A pilot test of oncological practices showed that complete family history report rates of 77% in FDRs and 61% in second-degree relatives, but age at diagnosis was recorded in only one-third of them (8). In order to benefit from results of this study, detailed family history of close relatives (father, brother, and son) would be needed along with the age at diagnosis of prostate cancer in them (<60, 60-69, ≥70 years), which is of course easier to obtain than the exact age at diagnosis for each affected family member.

The American Society of Clinical Oncology guideline also recommends that emphasis on careful documentation of the family history of close relatives (9). Self-reports can still serve as an important source of information in clinical counselling since only information of close relatives would be needed. For countries with mature registry (or electronic health record) system like Sweden, family history could be technically taken from register-based datasets although to our knowledge this is not the case in any country. A recent practice improvement project at Mayo Clinic showed that enhanced family history screening was a low-cost but effective method for individualized cancer surveillance (10). Various tools for family history collection are being developed and systematic reviews have shown a 46-78% improvement in data collection (11, 12). As an attempt to alleviate the burden of physicians, health policy makers could remove the system barriers and facilitate the development and implementation of family history collection tools (e.g., by providing websites, software and/or apps to collect the necessary family history before the actual visit of men to the physician). Adequate health education to the lay population seems also very important for timely assessment of risk profile. Health economic studies are warranted to encourage insurances to cover cost of evidence-based personalized early screening in high-risk men.”

As an effort toward individualized screening, in contrast to the past one-size-fits-all strategy, based on findings of this study, we suggest to divide men with family history into five diversified groups to be able to propose more accurate screening and prevention interventions according to their risk levels. The goal of precision medicine and the utilization of family history in the disease risk assessment are highly aligned (12). Further researches are warranted about practicalities of implementing screening based on family history and comparison of cost-effectiveness of risk-adapted screening versus that of current age-based screening practice.”

*Discussion, second last paragraph, line 3:*

“Country-specific clinical guidelines need to recommend men to start prostate cancer screening at different ages based on their family history and age at diagnosis of prostate cancer in their families. Results of our study (summarized in Table 1) can be easily used by clinicians based on answers to following two simple questions:

1) How many of your close relatives (father, brothers or sons) have ever received a prostate cancer diagnosis?

2a) If the answer to first question is 1, then ask:

Was the relative diagnosed before age 60 years, between 60 and 69, or at 70 or older?

2b) If the answer to first question is 2 or more, then ask:

Was any of relatives diagnosed before age 60 years?

Reviewer #5 (Ola Bratt, Professor of Urology, Gothenburg University, Sweden):

Dear Dr Misra

This study provides new, clinically important, knowledge aand will be of interest for many specialities, but I'm not convinced that a general medical journal like PLoS Medicine is the best choice - a urology or cancer journal may be better.

Moreover, the authors should present their data on prostate cancer risk as they are, rather than as direct evidence for start ages for screening.

Finally, I should mention that I recently reviewed this manuscript for another journal (can't find my notes, though, and can't remember which one) and recommended similar changes as I do know, which means that the authors have not much revised their manuscript since then.

Yours sincerely

Ola Bratt

Comments

This is a large register-based cohort study on the influence of various combinations of family history (FH) of prostate cancer (PC) on the cumulative incidence of advanced/lethal PC. The analyses are well executed and the study provides clinically important, new knowledge, but the presentation of the results in the manuscript and the conclusions may be improved.

Major concerns:

* 1) The calculated cumulative risks of Stage III/IV PC are clinically relevant, not least when considering recommendations about the start age of PC screening for men with a FH of PC, but it is not justified to present the results as "risk-tailored starting ages for PCa screening". The only way to provide evidence-based screening start ages for men with a FH of PC is through screening trials. There is absolutely no ground for claiming that the 10- or 15-year cumulative incidence of Stage III/IV PC are directly associated with the optimal starting age. The authors may of course discuss the use of their results for individualising the start age of PC screening and conclude that their results are suitable for this, but the proposed starting ages should not directly be presented as results. The manuscript needs to be re-written accordingly. Further examples of inappropriate phrasing are in the title ("Risk-adapted starting age of screening…"), the Abstract's conclusion ("an important step toward evidence-based personalized PCa screening guidance"), at the end of page 3 ("we aimed to provide risk-adapted starting age of screening for relatives of patients with PCa"; this could be changed to something like "we aimed to provide XXX that may be used for risk-adapting the starting ages…"), page 15 ("first-hand evidence-based information on risk-adapted (personalized) starting age of PCa screening") and in the final conclusion ("this study provides novel information on risk-adapted starting age of screening").

*Response: Thank you for your comments. This approach of calculating risk-adapted starting age of cancers has already been applied to colorectal cancer and breast cancer (22-24). We have moved the Figures 1 and 2 to the front of tables and following sentences were accordingly modified in Title, Abstract, Methods, Introduction, Methods, and Discussion sections:*

*Title*:

“Starting age of screening in relatives of patients with prostate cancer: Evidence from nationwide register-based cohort study to supplement current screening recommendations”

*Abstract, Conclusions paragraph, line 2*:

“Our clinically relevant findings can be used for evidence-based personalized PCa screening guidance, …….”

*Introduction, last paragraph, last sentence*:

“…, we aimed to provide clinically relevant evidence to be used for risk-adapted starting ages of screening….”

*Methods, paragraph 4, line 10*:

“This approach of calculating risk-adapted starting age of cancers has already been already applied to some other cancers (22-24).”

*Discussion, paragraph 8, line 1*:

“……, which resulted in first-hand information that can be used for risk-adapted (personalized) starting age of PCa screening, …...”

*Discussion, last paragraph, line 1*:

“In conclusion, this study provides novel information that can be used for risk-adapted starting age of screening in first-degree relatives of patients with PCa based on the world’s largest nationwide register-based family-cancer datasets.”

* 2) The suggested starting ages should rather be presented as something like "The age at which the risk of stage III/IV and/or lethal PC is the same as for men in the general population aged 50 years (0,2%)." Moreover, these ages should be presented with 95% confidence intervals.

*Response: Following explanatory sentence were added to the beginning of last paragraph of Methods section and the phrase “starting ages” was revised to “age of reaching mass screening level of risk” when appropriate:*

“All our suggested risk-adapted starting ages of prostate cancer screening are based on the age at which the risk of stage III/IV and/or lethal PC reached the same risk level in men at benchmark starting age of mass screening in the general population.”

*Confidence intervals for the ages have been calculated and added to the Table 1, S1-S3 Tables. The following sentence was accordingly added to Methods, paragraph 4, line 12*:

“The 95% confidence intervals of age-specific10-year cumulative risk were calculated using the 2.5 and 97.5 percentiles of the bootstrap estimate distribution made by bootstrapping method (200 replications), from which confidence intervals of proposed starting ages (ages of reaching mass screening level of risk) were derived.”

* 3) Title: In addition to the above comment, also "Real world evidence" and "cohort" are misleading. "Cohort" may lead readers to believe that a cohort of screened men were observed. "Register study" may be better.

*Response: The term “Real world” was deleted and “register-based” study was added to the Title as following:*

“Starting age of screening in relatives of patients with prostate cancer: Evidence from nationwide register-based cohort study to supplement current screening recommendations.”

* 4) The limitations of the study are not well discussed. 4a) One is that the age of diagnosis, that is an important variable in the analysis, is highly dependent on when an individual's cancer is detected (localised or metastatic) and whether the cancer was detected after PSA testing or not (PSA testing confers up to 15 years lead time). A man's risk of PC is thus differently affected by a father diagnosed with a small low-grade PC after PSA-testing at age 70 versus a father diagnosed with metastatic disease at the same age. PSA-testing was introduced in Sweden in 1990 and became common in the late 1990s. This means that the age at diagnosis of relatives diagnosed before 1990 affects the PC risk differently than does the age at diagnosis of relatives diagnosed after year 2000.

*Response: Additional analysis by restricting the follow-up to period after year 1990 was conducted and the risk estimations were consistent. Following sentences were accordingly modified/added to Methods, Results, and Discussion sections:*

*Methods, paragraph 5, line 3*:

“A sensitivity analysis was conducted to compare results for period 1990-2015 (after introducing PSA testing) and for the whole study period, 1958-2015.”

*Results, paragraph 1, line 1:*

***“Sensitivity analysis***

A sensitivity analysis by calendar period at diagnosis comparing the results for the period 1990-2015 to the ones for the whole period (1958-2015) was conducted and the analysis showed consistent results (S2 Fig).”

*Discussion, paragraph 4, line 6:*

“It is possible that the age at diagnosis of cancer in family members could be affected by PSA screening, for which we had no data. No national population-based PSA screening program has ever been recommended in Sweden and organized PSA testing was implemented in parts of Sweden only since 2019 (6). Despite this, an additional sensitivity analysis was performed to compare risk estimations over different periods and the results were consistent. A recent study has shown that even family history of prostate borderline or in situ carcinoma (mostly screen-detected lesions) and their age at diagnosis are rather equally important and should be taken into account in counselling of patients and their relatives (7).”

* 4b) Another limitation is that the family history usually isn't revealed at many of the suggested start ages, so for instance supplementary table 2 describes a hypothetic scenario when it comes to the rows representing "Age at diagnosis of youngest relative" for brothers affected at 60-69 and > 70 years (few men aged 40 years have a brother aged over 60 years).

*Response: As we have shown in a paper on breast cancer (*[*https://doi.org/10.1002/cncr.32815*](https://doi.org/10.1002/cncr.32815)*), unlike the static method of family history assessment by traditional familial risk studies, where an affected relative diagnosed even after the diagnosis of the index person would be included, the dynamic method adopted in our analysis reflected the current family history profile for each men and the history only changed when a new family member was diagnosed. In the analysis based on dynamic definition of family history, all the number of cancer patients were real ones, only a hypothetical example was given in the footnote of each table to make it easier for readers to understand how to use the table. In the whole datasets, 36,902 men had at least a brother 15 years older than themselves, and 5,967 men had brother 20 years older. So, the hypothetical examples were also based on real data. To clarify this more, following sentences were accordingly modified in/added to Methods section:*

*Methods, paragraph 3, line 13*:

“This represents the actual change in the real-world when men mostly learn about the diagnosis of his close relatives at different time points of his life. For each year in the follow-up of individuals, we updated the family history profile for every index man, representing the real-time status (number and youngest age at diagnosis among relatives) of individuals with PCa diagnosed in his family. Occurrence of PCa in family members after the date of PCa diagnosis in the index man was not considered in familial risk calculations.”

* 4c) Further limitations include that many men with a FH of PC obtain PSA testing and are diagnosed with stage 2 disease that is subsequently cured, but from which the man would have died had it not been detected and treated in time.

*Response:* *Following sentences were accordingly modified/added to Discussion, paragraph 9, line 12*:

“The stage III/IV or lethal familial risk estimations could have been diluted when men with family history of PCa were diagnosed early due to actively seeking earlier PSA screening and did not die of PCa compared to men without family history, although a previous study has reported that familial aggregation of PCa is not due to screening habits shared in a family (21). Screening practice may change the reference group in calculation of familial relative risk of aggressive PCa (e.g., in terms of standardized incidence ratio). However, our results in terms of cumulative risk of aggressive PCa that has no reference group is not affected by screening practice and this explains the stable results over different periods in a sensitivity analysis that we conducted. The possible risk-adapted starting ages of screening for populations at different benchmark starting ages of screening were provided to fit in with countries that have different risk levels of PCa.”

Minor comments

* 5) Methods, page 4: The statement "The Swedish National Board of Health and Welfare does not recommend a countrywide population-based PSA screening program for men" is not supported by reference 1.

*Response: The following correct reference (20) has been cited for that sentence in Methods, paragraph 2, line 2:*

“Frånlund M. Prostate Cancer Screening: Outcomes and Risk Prediction. Doctoral Thesis from University of Gothenburg, Sahlgrenska Academy, 2019.”

* 6) Methods, page 4: The outcome was not the risk of late-stage/fatal prostate cancer. First, because occurrence of PC was the outcome, not the risk of this. Second, stage III includes poorly differentiated, localised PC, which is early-stage, not late-stage PC).

*Response: All phrases of “late-stage” have been revised to “stage III/IV” and following sentences were accordingly added to Methods paragraph 2, line 4*:

“……, we used the occurrence of stage III/IV or fatal prostate cancer as the outcome. The exposure of interest was having a history of PCa (regardless of TNM stage or cancer-specific death status) in first-degree relatives (FDRs: father, brother, or son). The outcome was either diagnosis of stage III/IV PCa at the time of first diagnosis (based on the American Joint Committee on Cancer, 8^th^ edition of cancer TNM staging) and/or death due to PCa (3). The death was considered due to PCa when PCa was reported as the main underlying cause of death. As data on prostate-specific antigen and Gleason score/grade group were not available in the Swedish Cancer Registry, in this study, only those with ‘T3-4, N0, and M0’ status were considered as stage Ⅲ prostate cancer. Those with ‘T1-2, N0, and M0’ status were not considered as “with outcome” unless they died of PCa. These mean that the cancer has grown outside the prostate, broken through the capsule (covering) of the prostate gland, and might have spread to the seminal vesicles (T3), or it has spread into other tissues next to the prostate, such as the urethral sphincter, bladder, rectum, and/or the wall of the pelvis (T4). It has not spread to nearby lymph nodes (N0) or elsewhere in the body (M0). Stage IV was defined as either ‘Any T, N1, and M0’ or ‘Any T, Any N, and M1’, which means that the cancer has spread to either nearby lymph nodes (N1) or elsewhere in the body (M1).”

* 7) Methods, page 4: Most readers are probably not familiar with the AJCC staging system, so I recommend that stage III/IV is described in terms of TNM, Gleason score and PSA.

*Response: As shown in the following sentences, definitions of stage III and stage IV were accordingly added to Methods, Statistical analyses subsection, paragraph 2, line 7*:

“The outcome was either diagnosis of stage III/IV PCa at the time of first diagnosis (based on the American Joint Committee on Cancer, 8^th^ edition of cancer TNM staging) and/or death due to PCa (3). The death was considered due to PCa when PCa was reported as the main underlying cause of death. As data on prostate-specific antigen and Gleason score/grade group were not available in the Swedish Cancer Registry, in this study, only those with ‘T3-4, N0, and M0’ status were considered as stage Ⅲ prostate cancer. Those with ‘T1-2, N0, and M0’ status were not considered as “with outcome” unless they died of PCa. These mean that the cancer has grown outside the prostate, broken through the capsule (covering) of the prostate gland, and might have spread to the seminal vesicles (T3), or it has spread into other tissues next to the prostate, such as the urethral sphincter, bladder, rectum, and/or the wall of the pelvis (T4). It has not spread to nearby lymph nodes (N0) or elsewhere in the body (M0). Stage IV was defined as either ‘Any T, N1, and M0’ or ‘Any T, Any N, and M1’, which means that the cancer has spread to either nearby lymph nodes (N1) or elsewhere in the body (M1).”

* 8) Results, 3rd sentence: The "static approach" has not been described in Methods. I assume this approach is incorrect, so why present results for this approach?

*Response: As stated in Discussion, the static approach is used traditionally by familial risk assessments studies. The explanation was given to show the difference between these two methods and the appropriateness of our dynamic approach for the purpose of this study. Following sentences were modified in or added to Methods to clarify that we did not use the biased static approach:*

“In this study, a dynamic definition of family history of PCa (and not the static approach) was used to consider the changes in family history over time. Both methods and the superiority of the dynamic approach for the purpose of our risk stratification study have been discussed in detail elsewhere (14).”

* 9) Page 8, 1st sentence under "Risk by age at diagnosis in relatives" is wrong. It should either be "The younger the age at diagnosis of PCa in FDRs, the higher the risk of..." OR

"Younger age at diagnosis of PCa in FDRs was associated with higher risk of...".

*Response: This sentence has been accordingly revised to:*

“Younger age at diagnosis of PCa in FDRs was associated with higher risk of stage III/IV or fatal PCa in other family members (Figure 1 and Figure 2).”

* 10) Page 9, 1st sentence: "rather comparable" is vague. Maybe the authors can substitute for a different term?

*Response: The words ‘rather comparable’ were revised to ‘quite similar’:*

“….and the results were quite similar.”

* 11) Discussion, end of 1st paragraph: RE "current guidelines, which are mostly based on the experts' opinion rather than evidence" - I'd say that the guidelines are based on the same kind of evidence that the present study provides, namely knowledge of an earlier age at onset of PC in men with a FH for PC, but by writing "current guidelines, which are mostly based on the experts' opinion rather than evidence", the authors imply that their own recommendations are strongly evidence-based, which they are not.

*Response: The statement “*mostly based on the experts' opinion*” has been deleted.*

*12) It is questionable whether the by the present study's authors' suggested highly variable starting ages are clinically more useful than the previous ones. Is it really realistic to produce clinical guidelines in which men with a FH of PC are stratified into 30 different groups with different recommended start ages?

*Response: Individualized screening strategy is the consensus of current guidelines because the one-size-fits-all approach is not appropriate. Our calculated risk-adapted starting age of screening are provided for different benchmark ages to fit in with different populations. It is worth mentioning that country-specific clinical guidelines need to include men with a family history of prostate cancer stratified into only 5 different groups (not 30 groups) with different recommended start ages. Following sentences were accordingly added to Discussion.*

*Discussion, paragraph 12, line 1:*

As an effort toward individualized screening, in contrast to the past one-size-fits-all strategy, based on findings of this study, we suggest to divide men with family history into five diversified groups to be able to propose more accurate screening and prevention interventions according to their risk levels. The goal of precision medicine and the utilization of family history in the disease risk assessment are highly aligned (12). Further researches are warranted about practicalities of implementing screening based on family history and comparison of cost-effectiveness of risk-adapted screening versus age-based screening practice.”

*Discussion, paragraph 13, line 3:*

“Country-specific clinical guidelines need to recommend men to start prostate cancer screening at different ages based on their family history and age at diagnosis of prostate cancer in their families. Results of our study (summarized in Table 1) can be easily used by clinicians based on answers to following two simple questions:

1) How many of your close relatives (father, brothers or sons) have ever received a prostate cancer diagnosis?

2a) If the answer to first question is 1, then ask:

Was the relative diagnosed before age 60 years, between 60 and 69, or at 70 or older?

2b) If the answer to first question is 2 or more, then ask:

Was any of relatives diagnosed before age 60 years?”

*13) Page 13: Reference 26 reports deaths within 25-30 years, not 30-40 years.

*Response: The values ’30-40’ have been revised to’25-30’.*

* 14) The guidelines referred to from ACP, ACS & AUA (refs 13, 15, 16) are old (2010 - 2013). There must be newer versions.

*Response: We rechecked the official websites of ACP, ACS and AUA again, the references cited by this paper were still the most recent ones:*

*ACP:* [*https://www.acponline.org/clinical-information/guidelines/comparative-guideline-tables*](https://www.acponline.org/clinical-information/guidelines/comparative-guideline-tables)

*ACS:* [*https://www.cancer.org/cancer/prostate-cancer/detection-diagnosis-staging/acs-recommendations.html#references*](https://www.cancer.org/cancer/prostate-cancer/detection-diagnosis-staging/acs-recommendations.html#references)

*AUA:* [*https://www.auanet.org/guidelines/prostate-cancer-early-detection-guideline*](https://www.auanet.org/guidelines/prostate-cancer-early-detection-guideline) *(This guideline was published in 2013 and reviewed in 2018. The statement regarding family history of prostate cancer stayed the same)*

*The following 2018 revised AUA guideline (25) was also cited accordingly:*

“H. Ballentine Carter PCA, Michael J. Barry, Ruth Etzioni, Stephen J. Freedland, Kirsten Lynn Greene, Lars Holmberg, Philip Kantoff, Badrinath R. Konety, Mohammad Hassan Murad, David F. Penson and Anthony L. Zietman. Early Detection of Prostate Cancer (2018) 2018 [Available from: <https://www.auanet.org/guidelines/prostate-cancer-early-detection-guideline>.”

**References used in the response letter**

(*Note: Reference numbers created by EndNote are different from those in the manuscript*)

1.	Benchimol EI, Smeeth L, Guttmann A, Harron K, Moher D, Petersen I, et al. The REporting of studies Conducted using Observational Routinely-collected health Data (RECORD) statement. PLoS Med. 2015;12(10):e1001885.

2.	Cancer registration: principles and methods. IARC Sci Publ. 1991(95):1-288.

3.	Amin MB, Edge S, Greene F, Byrd DR, Brookland RK, Washington MK, et al. AJCC Cancer Staging Manual. 8 ed: American Joint Committee on Cancer. Springer International Publishing; 2017. XVII, 1032 p.

4.	Barlow L, Westergren K, Holmberg L, Talback M. The completeness of the Swedish Cancer Register: a sample survey for year 1998. Acta Oncol. 2009;48(1):27-33.

5.	Brooke HL, Talback M, Hornblad J, Johansson LA, Ludvigsson JF, Druid H, et al. The Swedish cause of death register. Eur J Epidemiol. 2017;32(9):765-73.

6.	Rekommendationer om organiserad prostatacancertestning: Regionala cancercentrum i samverkan; 2020 [Available from: <https://www.cancercentrum.se/samverkan/vara-uppdrag/prevention-och-tidig-upptackt/prostatacancertestning/>.

7.	Xu X, Fallah M, Tian Y, Mukama T, Sundquist K, Sundquist J, et al. Risk of invasive prostate cancer and prostate cancer death in relatives of patients with prostatic borderline or in situ neoplasia: A nationwide cohort study. Cancer. 2020.

8.	Wood ME, Kadlubek P, Pham TH, Wollins DS, Lu KH, Weitzel JN, et al. Quality of cancer family history and referral for genetic counseling and testing among oncology practices: a pilot test of quality measures as part of the American Society of Clinical Oncology Quality Oncology Practice Initiative. J Clin Oncol. 2014;32(8):824-9.

9.	Lu KH, Wood ME, Daniels M, Burke C, Ford J, Kauff ND, et al. American Society of Clinical Oncology Expert Statement: collection and use of a cancer family history for oncology providers. J Clin Oncol. 2014;32(8):833-40.

10.	Clift K, Macklin SK, Wallace MB, Presutti RJ. Enhanced family history screening is crucial to individualized cancer surveillance. Journal of Clinical Oncology. 2019;37(15_suppl):e13161-e.

11.	Qureshi N, Carroll JC, Wilson B, Santaguida P, Allanson J, Brouwers M, et al. The current state of cancer family history collection tools in primary care: a systematic review. Genet Med. 2009;11(7):495-506.

12.	Ginsburg GS, Wu RR, Orlando LA. Family health history: underused for actionable risk assessment. Lancet. 2019;394(10198):596-603.

13.	Hemminki K, Sundquist J, Bermejo JL. How common is familial cancer? Ann Oncol. 2008;19(1):163-7.

14.	Mukama T, Kharazmi E, Sundquist K, Sundquist J, Brenner H, Fallah M. Familial risk of breast cancer by dynamic, accumulative, and static definitions of family history. Cancer. 2020.

15.	Bray F, Ferlay J, Soerjomataram I, Siegel RL, Torre LA, Jemal A. Global cancer statistics 2018: GLOBOCAN estimates of incidence and mortality worldwide for 36 cancers in 185 countries. CA Cancer J Clin. 2018;68(6):394-424.

16.	Jonsson H, Holmstrom B, Duffy SW, Stattin P. Uptake of prostate-specific antigen testing for early prostate cancer detection in Sweden. Int J Cancer. 2011;129(8):1881-8.

17.	Enblad AP, Bergengren O, Andren O, Larsson A, Fall K, Johansson E, et al. PSA testing patterns in a large Swedish cohort before the implementation of organized PSA testing. Scand J Urol. 2020:1-6.

18.	Nordstrom T, Aly M, Clements MS, Weibull CE, Adolfsson J, Gronberg H. Prostate-specific antigen (PSA) testing is prevalent and increasing in Stockholm County, Sweden, Despite no recommendations for PSA screening: results from a population-based study, 2003-2011. Eur Urol. 2013;63(3):419-25.

19.	Bevier M, Weires M, Thomsen H, Sundquist J, Hemminki K. Influence of family size and birth order on risk of cancer: a population-based study. BMC Cancer. 2011;11:163.

20.	Frånlund M. Prostate Cancer Screening: Outcomes and Risk Prediction [Doctoral thesis]. Gothenburg, Sweden: University of Gothenburg; 2019.

21.	Brandt A, Sundquist J, Hemminki K. Risk for incident and fatal prostate cancer in men with a family history of any incident and fatal cancer. Ann Oncol. 2012;23(1):251-6.

22.	Mukama T, Kharazmi E, Xing X, Sundquist K, Sundquist J, Brenner H, et al. Risk-Adapted Starting Age of Screening for Relatives of Patients With Breast Cancer. JAMA Oncol. 2019.

23.	Tian Y, Kharazmi E, Brenner H, Xu X, Sundquist K, Sundquist J, et al. Calculating the Starting Age for Screening in Relatives of Patients With Colorectal Cancer Based on Data From Large Nationwide Data Sets. Gastroenterology. 2020;159(1):159-68 e3.

24.	Mukama T, Fallah M, Tian Y, Sundquist K, Sundquist J, Brenner H, et al. Risk-tailored starting age of breast cancer screening based on women's reproductive profile: A nationwide cohort study. Eur J Cancer. 2020;124:207-13.

25.	H. Ballentine Carter PCA, Michael J. Barry, Ruth Etzioni, Stephen J. Freedland, Kirsten Lynn Greene, Lars Holmberg, Philip Kantoff, Badrinath R. Konety, Mohammad Hassan Murad, David F. Penson and Anthony L. Zietman. Early Detection of Prostate Cancer (2018) 2018 [Available from: <https://www.auanet.org/guidelines/prostate-cancer-early-detection-guideline>.
