# Supplementary material for: Risk of prostate cancer in relatives of prostate cancer patients in Sweden: A nationwide cohort study
Source: PLoS Med. 2021 Jun 1;18(6):e1003616. doi: 10.1371/journal.pmed.1003616 (PMC8168897; doi:10.1371/journal.pmed.1003616)
Supplement: Attachment — Submitted filename: Final Reponse letter 4Dec2020.docx [file pmed.1003616.s010.docx]

**Requests from the editors:**

*Title: study descriptor needed, remove starting age of screening etc. I suggest “Risk of cancer in relatives of patients with Prostate Cancer in Sweden: A cohort study” or similar

*Response: The title was revised to*:

“Risk of prostate cancer in relatives of patients with this cancer in Sweden: A nationwide cohort study”

* Abstract- aim reads more like a recommendation. Need to revise this. There are recurring mentions of “providing risk adapted age of screening” however, I wonder if this is an implication of your findings and the work highlights the adapted risk of prostate cancer instead? Please revise throughout, as needed.

*Response: This phrase was revised throughout.*

*Abstract, Background section, third line*:

“We aimed to provide evidence for risk-adapted starting age of screening for relatives of patients with PCa”

*Abstract, Conclusions section, first line*:

“Our study provides practical information for risk-tailored starting ages of PCa screening…”

* Abstract- age range of participants

*Response: The age range was accordingly added to Abstract, Method and Findings section, first line*:

“In this register-based nationwide cohort study, all men (aged 0 to 96 years at baseline) residing in Sweden who were born after 1931……”

*It was also added to Results section, first line*:

“During the follow-up of 6,343,727 men (aged 0 to 96 years at baseline), a total of 88,999 patients were diagnosed with stage III/IV PCa or died due to PCa.”

* Please add 2-3 limitations of your study in the last sentence of the abstract

*Response: The following sentence was accordingly added to Abstract, Method and Findings section, last line*:

“No genetic data or information on lifestyle was available. Parents’ ages were not limited but offspring were followed up to age 84 years. Completeness of cancer registry was about 96%.”

* Abstract conclusions- I suggest revising “highly valid”

*Response: The word “highly-valid” was removed and the sentence now reads as following:*

“Our study provides practical information for risk-tailored starting ages of PCa screening based on nationwide cancer data with valid genealogical information.”

* Data availability- please add contact details of the relevant person(s) at Lund University who may be able to organise access to data for third parties

*Response: The following Data availability statement including relevant contact details will be submitted as metadata in the online submission system*:

“**Data Availability**: This study made use of the Swedish Cancer Registry data. Data from the nationwide registers cannot be shared by study authors, however further information and relevant contact details can be found on: <https://www.socialstyrelsen.se/en/statistics-and-data/registers/register-information/>

Postal address: Socialstyrelsen, SE-106 30 Stockholm, Sweden

Phone: +46 (0)75 247 30 00

Fax: +46 (0)75 247 32 52

E-mail: [socialstyrelsen@socialstyrelsen.se](mailto:socialstyrelsen@socialstyrelsen.se)

Links for Swedish Cancer Registry: <https://www.socialstyrelsen.se/en/statistics-and-data/registers/register-information/swedish-cancer-register/>

Email address: [cancerregistret@socialstyrelsen.se](mailto:cancerregistret@socialstyrelsen.se)”

* The author summary section “what did the researchers do” does not provide a summary of the research methods but its implementation in the clinic. Please remove the second and third bullet point and provide a summary of the methods and results

*Response: The second, third and fourth bullet points were accordingly revised or removed. A summary of the methods and results was added*:

- “This study estimated the risk of stage III/IV or fatal prostate cancer in close family members of patients with prostate cancer.
- For each single age, we calculated the risk of developing aggressive PCa in the next 10 years; for example, at age 50, which is a common age for starting PCa screening, the risk of developing PCa during next 10 years (age 50 to 59) in the Swedish population was 0.2%.
- Men with family history of PCa reach this screening risk thresholds, up to 12 years earlier than the general population.
- We also calculated risk-adapted estimates for other starting ages of screening in the guidelines, such as 45, 55, and 60 years and compared our findings with those in the common guidelines.
- This study found that age, age at diagnosis of PCa in relative/s, and number of affected FDRs are important elements in increased risk of stage III/IV or fatal PCa and these factors accordingly resulted in different risk-adapted starting ages of PCa screening.
- The results showed that relatives of patients with PCa reached the screening level of risk up to 11 years earlier than the recommended starting ages of PCa screening by current guidelines.”

* Please use quare brackets for references throughout

*Response: The brackets of references were all changed to square brackets.*

* Please provide the analysis plan as supplementary information and add a call out to this document in the methods section.

*Response: The analysis plan was added as supporting information and was cited in Method and Findings section, last paragraph, line 9*:

“The analyses were planned ahead and no data-driven changes to analyses took place (S1 Analysis plan).”

* In addition, please provide a completed RECORD checklist as supplementary information, using paragraphs and sections instead of page numbers

*Response: A completed and updated RECORD checklist was added as supporting information (S1 RECORD checklist).* *Page numbers were replaced with section name and paragraph numbers.*

* Page 12 “of PCa screening by current guidelines, which are mostly based on the experts’ opinion rather than evidence” this statement comes off rather combative and I suggest removing or rephrasing. Please also add a citation to the current guidelines, noting whether they are national (Swedish) or international guidelines

*Response: This sentence was removed and the following citations were added to “the current guidelines”*:

“…….current guidelines in North America and Europe [1-8].”

* I suggest replacing all iterations of “high quality data” in the main text to be replaced by nationwide data or similar

*Response: All the iterations were revised to “nationwide data” or removed.*

* I suggest organizing the Discussion as follows: a short, clear summary of the article's findings; what the study adds to existing research and where and why the results may differ from previous research; strengths and limitations of the study; implications and next steps for research, clinical practice, and/or public policy; one-paragraph conclusion. The discussion is rather long and perhaps needs subheadings for readability.

*Response: Thank you for your suggestions. The Discussion section was organized as suggested and following subheading were added to this section*:

***“Findings of the study***

***Comparisons with other studies***

***Strengths and limitation***

***Implications of findings***

***Conclusion”***

* Page 14 “current experts’ opinion-based recommendations for screening in relatives of patients with PCa are already in practice in many countries around the world without validation”. I suggest adding a reference or two in support or removing this sentence.

*Response: This sentence was removed.*

**Comments from the reviewers:**

Reviewer #4: NO further comments

*Response: Thank you.*

Reviewer #5:

* The authors have extensively revised and much improved the manuscript in accordance with the reviewers' numerous comments and suggestion. I must complement them on their thorough consideration of all the comments.

I have only one small suggestion, which the authors can feel free to adjust to or to discard, about their resopnse to my comment 5 Methods, page 4:

The statement "The Swedish National Board of Health and Welfare does not recommend a countrywide population-based PSA screening program for men" is probably better supported by the following reference than by the dissertation.

Screening för prostatacancer. Rekommendation och bedömningsunderlag. Socialstyrelsen 2018. Access at <https://www.socialstyrelsen.se/globalassets/sharepoint-dokument/artikelkatalog/nationella-screeningprogram/2018-10-15.pdf>

*Response: Thank you for your suggestion. We have replaced the reference with the one from ‘Socialstyrelsen’.*

**References used in the response letter**

(*Note: Reference numbers created by EndNote are different from those in the manuscript*)

1.	Force USPST, Grossman DC, Curry SJ, Owens DK, Bibbins-Domingo K, Caughey AB, et al. Screening for Prostate Cancer: US Preventive Services Task Force Recommendation Statement. JAMA. 2018;319(18):1901-13.

2.	Mottet N, Bellmunt J, Bolla M, Briers E, Cumberbatch MG, De Santis M, et al. EAU-ESTRO-SIOG Guidelines on Prostate Cancer. Part 1: Screening, Diagnosis, and Local Treatment with Curative Intent. Eur Urol. 2017;71(4):618-29.

3.	Qaseem A, Barry MJ, Denberg TD, Owens DK, Shekelle P, Clinical Guidelines Committee of the American College of P. Screening for prostate cancer: a guidance statement from the Clinical Guidelines Committee of the American College of Physicians. Ann Intern Med. 2013;158(10):761-9.

4.	Rendon RA, Mason RJ, Marzouk K, Finelli A, Saad F, So A, et al. Canadian Urological Association recommendations on prostate cancer screening and early diagnosis. Can Urol Assoc J. 2017;11(10):298-309.

5.	Wolf AM, Wender RC, Etzioni RB, Thompson IM, D'Amico AV, Volk RJ, et al. American Cancer Society guideline for the early detection of prostate cancer: update 2010. CA Cancer J Clin. 2010;60(2):70-98.

6.	Carter HB, Albertsen PC, Barry MJ, Etzioni R, Freedland SJ, Greene KL, et al. Early detection of prostate cancer: AUA Guideline. J Urol. 2013;190(2):419-26.

7.	Carroll PR, Parsons JK, Andriole G, Bahnson RR, Castle EP, Catalona WJ, et al. NCCN Guidelines Insights: Prostate Cancer Early Detection, Version 2.2016. J Natl Compr Canc Netw. 2016;14(5):509-19.

8.	H. Ballentine Carter PCA, Michael J. Barry, Ruth Etzioni, Stephen J. Freedland, Kirsten Lynn Greene, Lars Holmberg, Philip Kantoff, Badrinath R. Konety, Mohammad Hassan Murad, David F. Penson and Anthony L. Zietman. Early Detection of Prostate Cancer (2018) 2018 [Available from: <https://www.auanet.org/guidelines/prostate-cancer-early-detection-guideline>.
