# Supplementary material for: Risk of prostate cancer in relatives of prostate cancer patients in Sweden: A nationwide cohort study
Source: PLoS Med. 2021 Jun 1;18(6):e1003616. doi: 10.1371/journal.pmed.1003616 (PMC8168897; doi:10.1371/journal.pmed.1003616)
Supplement: Attachment — Submitted filename: MF2 XX R3 Reponse letter 6Jan2021.docx [file pmed.1003616.s011.docx]

**Requests from the editors:**

*Please amend the title to "Risk of prostate cancer in relatives of prostate cancer patients in Sweden ..."

*Response: The title was revised to*:

“Risk of prostate cancer in relatives of prostate cancer patients in Sweden: A nationwide cohort study”

* Please begin the abstract "Evidence-based guidance ..."

*Response: The beginning of the Abstract was revised to*:

“Evidence-based guidance…”

* In the abstract, please quote 95% CI around observations such as "9 years earlier" (you may prefer to state the age with associated 95% CI, as in table 1)

*Response: The 95% CIs for ages were added.*

“Men with ≥2 FDRs diagnosed with PCa reached this screening level at age 41 (95% CI: 39-44), i.e. 9 years earlier, when the youngest one was diagnosed before age 60; at age 43 (41-47), i.e. 7 years earlier, when ≥2 FDRs were diagnosed after age 59, which was similar to that of men with one FDR diagnosed before age 60 (41-45); and at age 45 (44-46) when one FDR was diagnosed at age 60-69, and 47 (46-47) when one FDR was diagnosed after age 69.”

* In the abstract and elsewhere, please write "9 years earlier" rather than "nine years earlier", and so on, except at the start of sentences.

*Response: The “nine/seven/five/three years earlier” were accordingly revised to “9/7/5/3 years earlier” throughout the manuscript*.

* Please revisit the phrase "parents' ages were not limited ..." in the abstract, which may need a few words of explanation - do you mean that full information on participants' fathers' lifespans was available in the study?

*Response: Yes, the full information on participant’ fathers’ lifespans was available. However, to avoid confusion, this part of the sentence was removed from Abstract and a similar sentence in the* *Methods section (Paragraph 1, line 7) was modified to:*

“In this nationwide cohort study, full information on participants' fathers' lifespans (if not restricted by beginning/end of cancer registry data or immigration/emigration) was available in the study but offspring were followed only up to age 84 years.”

* Please add a new final sentence to the "methods and findings" subsection of your abstract, which should begin "Study limitations include ..." or similar, and should list 2-3 of the study's main limitations.

*Response: The following sentence was added*:

“Study limitations include the lack of genetic data, information on lifestyle, and external validation.”

* In the "Conclusion" subsection of your abstract, please adapt "can be used ..." to "could be used ...", to avoid overstatement of your findings' relevance in other populations. Please make a similar change in the final paragraph of the main text.

*Response: This phrase in the abstract and similar expressions in the main text were revised accordingly*:

*Abstract, Conclusions section, third line*:

“…findings could be used for…”

*Discussion, paragraph 4, last sentence*:

“…, which could be used in …”

*Discussion, paragraph 7, second line*:

“…information that could be used for …”

*Discussion, Conclusion section, first line*:

“…that could be used for …”

* Please delete the "keywords" section from the abstract page.

*Response: The “Keywords” section has been deleted.*

* In the author summary, please spell out "FDR" and "PCa" at first use.

*Response: The abbreviations were spelled out at first use.*

* Please trim the central subsection of your author summary, which should consist of no more than 3-4 points of 1-2 short sentences each.

*Response: The central subsection of the Author Summary was revised to following 4 points*:

- In this nationwide study on 6,343,727 men, the risk of stage III/IV or fatal PCa in close family members of patients with PCa was estimated.
- It was observed that men with family history of PCa reach the screening risk threshold up to 12 years earlier than the general population.
- This study found that age, age at diagnosis of PCa in relative/s, and number of affected first-degree relatives are important elements in increased risk of stage III/IV or fatal PCa and these factors accordingly resulted in different risk-adapted starting ages of PCa screening.
- Comparison between our evidence-based risk-adapted starting age of screening and recommended age of PCa screening by different guidelines showed a difference ranging from -2 to 11 years.

* Please avoid "parent/s" in the author summary and any other instances in the paper.

*Response: The term parent has been revised to “father” when applicable.*

* In the author summary, please amend the text to "... reach this screening risk threshold up to 11 years earlier ...".

*Response: This text was revised as suggested.*

* We notice that the author summary includes statements of "up to 11 years earlier" and "up to 12 years earlier". Please correct any inconsistency here or elsewhere in the paper.

*Response: Thank you very much for your comment. "up to 11 years earlier" refers to the age gaps when compared with different benchmark starting ages of screening in the population, whereas. “up to 12 years earlier” refers to the age gaps when compared with the recommended ages of PCa screening by current guidelines. To make it clearer, we modified the last point of middle section of Author summary to:*

“Comparison between our evidence-based risk-adapted starting age of screening and recommended age of PCa screening by different guidelines showed a difference ranging from -2 to 11 years.”

* Please revisit the statement "the world's largest data" in your author summary. We suggest amending this to "Our study made use of the largest dataset available, to our knowledge, to identify the optimal age ..." or similar.

*Response: This statement was revised to:*

*“*This study made use of the largest dataset available, to our knowledge, to identify the optimal age for starting PCa screening in relatives of patients with PCa*”.*

* On p.14 of the paper, please make that "... we used the largest familial cancer datasets available, to our knowledge, and in our analyses considered ...".

*Response: This sentence was revised to*:

“…we used the largest familial cancer datasets available, to our knowledge, and in our analyses considered…”

* Please revisit the "Strengths and weaknesses" subsection in the discussion. We suggest sumarizing the study's possible weaknesses in a discrete paragraph in a neutral way. At present, interspersed with statements such as "another important advantage", the discussion seems to avoid acknowledging possible limitations and implications for the study's conclusions. For example, are there possible differences in groups of different ethnicity?

*Response: The sentences with statements such as "another important advantage" were moved to the discrete paragraph for strengths. We summarized the study's possible weaknesses in a discrete paragraph in the Discussion (subsection Strengths and weaknesses, Paragraph 2) and stressed the lack of ethnicity-based analysis as a limitation at the end of this paragraph*:

“We had no data on genetic information, however, family history, which represents familial risk stemming from shared genetic and shared environmental factors, is still the strongest risk factor for PCa that plays an important role in risk evaluation and decision-making about initiation of PCa screening (1-3)*.* The level of screening affects the proportion of men with a family history, which in turn would affect the proportion of men having to be screened at earlier ages. It could also affect the excess risk of stage III/IV or fatal cancer associated with a family history, since with screening a higher proportion of cases in relatives would be indolent. […] Another limitation of this study was the rather homogeneity of Swedish population, which did not allow stratified analyses by race/ethnicity. However, as different starting ages of screening are (or should be) recommended for different races/ethnicities or countries, the possible risk-adapted starting ages of screening for populations at different benchmark starting ages of screening were provided to fit in with races/ethnicities/countries that have different risk levels of PCa.”

* Throughout the text, please remove spaces from the reference call-outs (i.e., "... of Sweden [47,48].").

*Response: The spaces from the reference call-outs were all removed.*

* Please delete the "author contributions" section from the end of the main text.

*Response: The "Author contributions" section was deleted.*

* Please spell out the institutional author name for reference 9.

*Response: The institutional author name was spelled out.*

* Noting references 26, 28 & 45, for example, please ensure that all citations contain full access details.

*Response: The access details were added.*

* We did not find the RECORD checklist with your revised ms. Please ensure that this is available as a supplementary file ("S1_RECORD_Checklist" or similar) with your next revision; in the checklist, individual items should be referred to by section (e.g., "Methods") and paragraph number rather than by line or page numbers, as the latter generally change in the event of publication.

*Response: A completed and updated RECORD checklist was added as a Supporting information (S1 RECORD checklist).* *Page numbers were replaced with section name and paragraph numbers.*

**Comments from the reviewers:**

Reviewer #4: NO further comments

*Response: Thank you.*

Reviewer #5:

* The authors have extensively revised and much improved the manuscript in accordance with the reviewers' numerous comments and suggestion. I must complement them on their thorough consideration of all the comments.

I have only one small suggestion, which the authors can feel free to adjust to or to discard, about their resopnse to my comment 5 Methods, page 4:

The statement "The Swedish National Board of Health and Welfare does not recommend a countrywide population-based PSA screening program for men" is probably better supported by the following reference than by the dissertation.

Screening för prostatacancer. Rekommendation och bedömningsunderlag. Socialstyrelsen 2018. Access at <https://www.socialstyrelsen.se/globalassets/sharepoint-dokument/artikelkatalog/nationella-screeningprogram/2018-10-15.pdf>

*Response: Thank you for your suggestion. We have replaced the reference with the one from ‘Socialstyrelsen’.*

**References used in the response letter**

(*Note: Reference numbers created by EndNote are different from those in the manuscript*)

1.	Attard G, Parker C, Eeles RA, Schroder F, Tomlins SA, Tannock I, et al. Prostate cancer. Lancet. 2016;387(10013):70-82.

2.	Gronberg H. Prostate cancer epidemiology. Lancet. 2003;361(9360):859-64.

3.	Hemminki K. Familial risk and familial survival in prostate cancer. World J Urol. 2012;30(2):143-8.
